# Tracing the Right Path: Determination of Large Pedigree Segmentation and Relatedness

**DOI:** 10.1007/s10519-026-10259-z

**Published:** 2026-06-01

**Authors:** Michael D. Hunter, S. Mason Garrison, Xuanyu Lyu, Rachel Good, M. Nithya Mylakumar, Lihle Bayavuya Moyakhe, Sarah L. Carroll, S. Alexandra Burt

**Affiliations:** 1 Department of Human Development and Family Studies, Pennsylvania State University, 133 Health and Human Development Building, University Park, PA 16802 USA; 2 Wake Forest University, Winston-Salem, USA; 3 University of Colorado at Boulder, Boulder, USA; 4 Michigan State University, East Lansing, USA

**Keywords:** Population database, Path tracing, Software, Pedigree data

## Abstract

Large population databases frequently include complicated family structures that are amenable to modern biometric methods: allowing for intergenerational and extended pedigree analyses. To date, much of the latent potential of these resources remains untapped due to numerous complexities that arise in their analysis. Two difficult and critical problems are (1) finding independent extended families within larger population databases, and (2) determining coefficients of relatedness among all pairs of individuals within those extended families. If these problems were solved, researchers could more fully utilize data on extended families for biometric modeling. In this paper, we provide fast, computationally efficient algorithms for both of these problems and several more that are applicable to arbitrarily large and complex pedigrees. The algorithms rely solely on mother-child and father-child relationships that form the basis of many large population databases. These methods will be invaluable to any researcher trying to segment standard pedigree data files into independent extended family units, compute relatedness coefficients within extended families, and conduct intergenerational and other biometric modeling.

## Introduction

Several countries maintain large and complex databases of extended families, often hidden within national registries, rich with information on their populations. These national data resources are often designed for several purposes simultaneously: ranging from tracking economic and educational indicators, to fertility and health-related matters. Because information on child parentage is both readily available and useful for many purposes, it is a nearly ubiquitous feature of many national data resources. Yet the richness of this parental information remains substantially untapped (Stefansdottir et al. 2012; Almasy 2022). The present paper describes two critical challenges that prevent further use of this parental information in large databases of extended families, and moreover, provides algorithmic solutions to these challenges.

The first challenge is parsing large databases into smaller extended families. For the purposes of this paper, we define an extended family as a set of people connected by parentage, no matter how remotely. For example, grandchildren are connected to grandparents as the grandchildren’s parents’ parents’. Similarly, distant cousins can trace their lineage back through common parentage several generations in the past. In short, an extended family is any set of people that have any degree of familial connection to one another. This broad definition justifies people in distinct extended families being analyzed independently. Because statistical independence is a key feature of many data analysis procedures, finding independent units of analysis is a critical preparatory step. The independent units of analysis are extended families. In essence, this first challenge is to transform a single, large database that is too unruly to analyze into a set of smaller, more manageable databases that can be analyzed separately. The reduction in size from the entire database to the independent, extended families will be determined by the structure of the population and the design of the database. In some scenarios it is entirely possible that the database will be composed of a single, large, extended family. Databases that result from small, founder populations are one such example. Speculatively, it is possible that completely recorded populations with unrestricted mating might show a similar pattern. However, many databases are purposefully incomplete (e.g., collected through probability sampling) or have restricted mating patterns (e.g., in animal breeding and in some aristocratic families). In these latter cases, the database will be composed of numerous extended families across a range of sizes.

The second challenge is quantifying the nature of the relationship between all pairs of individuals within an extended family. Understanding that two people are related is of limited value without a detailed qualitative or quantitative description of that relationship. Qualitatively, relationship names describe the relationship between two individuals. For example, a “second cousins once removed” relationship occurs when one person is the grandchild of the other’s great-grandparent (or the child of the other’s second cousin). Quantitatively, a numeric index describes the same relationship path (e.g., .015625 for second cousins once removed). Although several forms of quantification are possible, the additive relatedness coefficient is by far the most useful to employ. The relatedness coefficient is the expected proportion of shared segregating genes between two individuals. For instance, parents and their children, much like full siblings and dizygotic twins, share on average 50% of their segregating genes. Half-siblings share about 25% of these genes, and first cousins share about 12.5%. In simple, highly-structured family designs, all of these relatedness coefficients are trivial to obtain; however, in large population databases, the family structures are so complex and varied that relatedness coefficients spanning even three or four generations are nigh impossible to compute with standard methods.

A large number of biometric models rely on knowing the coefficient of expected, additive, nuclear DNA relatedness for all pairs of individuals in a study. When paired with information about other coefficients of relatedness (e.g., sharing gestational environment, or sharing a child rearing environment), the expected proportion of shared segregating genes facilitates a variety of biometric models that tease apart environmental and genetic sources of variability in studied phenotypes. The absence of these relatedness coefficients impedes further biometric modeling on these valuable data resources.

Because of the intricate, varied nature of family relationships and the extensive diversity in family tree structures found across different populations, simple closed-form solutions for computing coefficients of expected relatedness do not exist. Prior research has relied on brute force solutions with narrow scopes (e.g., a single city; Moreau et al. 2011), or simplified approximations with numerous assumptions (Vigeland 2020) that do not account for the full complexity of extended family networks. Other methods have directly used measured genetic data (e.g., Identical By Descent (IBD) segment-based approaches; VanRaden 2008), but these have been shown to be effective only up to the second degree of relatedness and often struggle with accurately determining relationships beyond second cousins (Ramstetter et al. 2017).

Two notable exceptions to the rule of simple and limited relatedness computation are worth specific mention. The first exception is the work of Kaplanis et al. (2018) which calculated relatedness coefficients for millions of pairs of people in complex family structures, yet even this work relied on publicly posted family trees, which are not economical to scale. The second exception is a long history of pedigree computations in animal breeding (Wright 1922; Emik and Terrill 1949; Quaas 1976). We update and extend this literature to the context of human populations and modern computational methods, noting parallels where relevant.

Beyond these primary challenges of segmentation and quantification, there are additional concerns with large population databases such as data quality and the presence of partially missing records. However, solutions to these two challenges cascade into solutions for several closely related problems. A procedure for finding separate extended families can be immediately generalized to find separate matrilineal or patrilineal lines as well. Similarly, a procedure for quantifying additive genetic relatedness can be extended to other kinds of heritable patterns. Furthermore, this same procedure can algorithmically organize an extended family into hierarchical generations and compute the degree of relatedness between individuals (i.e., the path length in a drawn pedigree diagram). Thus, the present paper shows that the three identification variables that are most common in large population databases (child ID, mother ID, and father ID) are sufficient to:

1. Find independent genetic lines spanning multiple generations (i.e., extended families)
2. Find separate matrilineal lines and patrilineal lines
3. Compute the degree of relatedness and the appropriate generation number
4. Determine all coefficients of relatedness across an entire database

Moreover, we provide an implementation of algorithms that solve these problems in the BGmisc package (Garrison et al. 2024) in R (R Core Team, 2023). Although other researchers (e.g., Kaplanis et al. 2018) have computed relatedness coefficients for large and complex pedigrees, the present work relies on an absolutely minimal amount of information – only parentage – to achieve the same goal while simultaneously solving a set of closely related problems.

A related challenge of high practical importance that these procedures solve occurs when populations experience decreases in genetic diversity. Disease, migration patterns, and highly selective breeding patterns (e.g., in animal breeding; VanRaden, 2008) often create genetic bottlenecks that lead to higher amounts of relatedness than simple breeding models suggest amongst both classically-related and classically-unrelated individuals. Small founder populations are a classic example of precisely this bottleneck effect. Because the procedures outlined and implemented in this paper are sufficiently general to encompass virtually any breeding pattern, they simultaneously account for these deviations from asymptotic limits of large breeding populations.

The remainder of this paper is structured as follows. First, we use a small running example to illustrate each of the four problems. Second, we describe algorithms that solve each of the four problems. Third, we apply these solutions to more intricate examples that would be extremely difficult to solve by hand, but yield easily to these algorithms.

## Small Example

**Fig. 1:**
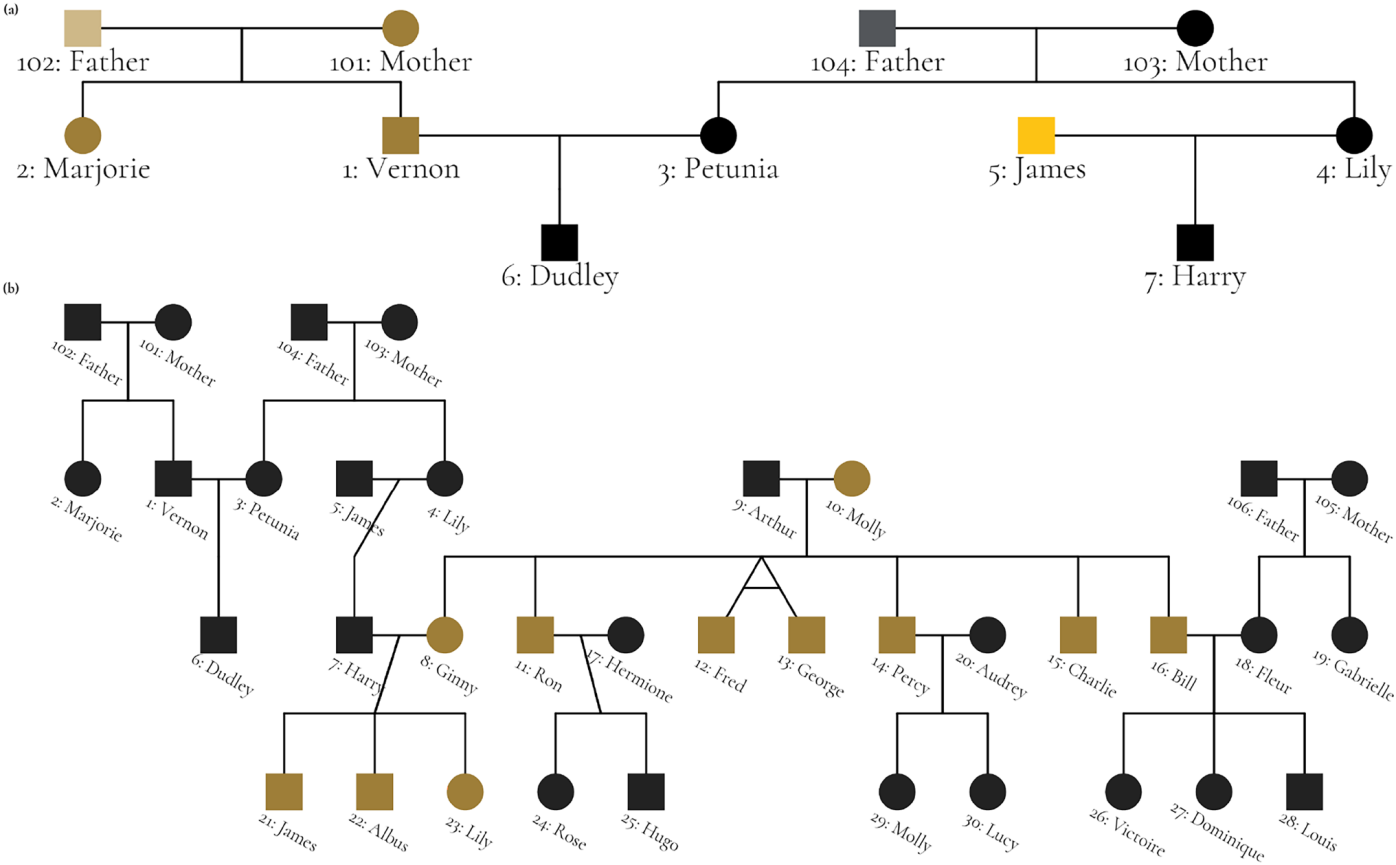
Small example pedigree diagrams generated with ggpedigree. Squares indicate males; circles indicate females. Joining lines horizontally indicate procreation. Joining down indicates descent. Unknown parental identifiers are replaced with “Mother” and “Father” as appropriate. **a** A 7-person pedigree with pseudo-nodes for parents that are not fully known; node shading distinguishes multiple maternal lines **b** A 30-person pedigree that extends panel (a) with an additional generation and their inter-relations. Shading highlights the largest maternal line (i.e., the maternal line originating from Molly)

Consider a small example dataset where we begin by having data on exactly one pair of siblings: ID 1 and ID 2. For the sake of convenience, we give the IDs names instead of numbers. ID 1 is a brother called Vernon; ID 2 is a sister called Marjorie. We know these people are full siblings but do not have full information about their parents. Continuing to look through the data we find we have a spouse for Vernon named Petunia. Furthermore, Petunia has a sister named Lily, and Lily has a spouse named James. So far, in this dataset we have five total people: two pairs of full siblings and their spouses. The pedigree structure implied is quite simple; it is easy to arrange by hand; it is easy to write computational algorithms that detect and solve all the possible relationships in this structure. Figure 1 (a) – created with the ggpedigree (Garrison 2025, 2026) package in R – shows a slightly extended 7-person pedigree along with pseudo-nodes for the parents of our primary IDs and a child for each of the spousal pairs. All possible relationships in this pedigree are relatively clear with minimal effort.

However, when we suppose that each of the spousal pairs has children, it opens the possibility that one of those children marries into a family of seven siblings, and that at least some of these children have children of their own. Very quickly we have a single extended family with 30 people that spans three generations with complex genealogical – not to mention interpersonal – relationships. Figure 1 (b) shows this full 30-person pedigree. Although still tractable, many relationships – especially more distant ones – are far less clear than in the 7-person pedigree.

One can easily imagine extending the 30-person pedigree of Fig. 1 to the kinds of data commonly encountered in large population databases. In these databases, it is common to have four or more generations on tens of thousands or hundreds of thousands of individuals. These cases are far beyond the reach of simple by-hand path tracing rules and visual inspection of a pedigree diagram. The complexity is compounded by the standard format of many large pedigree databases which only contain a person ID, mother ID, and father ID. All sibling, cousin, and other extended relationships must be inferred from this limited information. Table 1 gives information on our small pedigree in the standard format found in large population databases.

**Table 1:** Small pedigree dataset

ID	MaID	PaID	Name	ID	MaID	PaID	Name
1	101	102	Vernon	16	10	9	Bill
2	101	102	Marjorie	17	NA	NA	Hermione
3	103	104	Petunia	18	105	106	Fleur
4	103	104	Lily	19	105	106	Gabrielle
5	NA	NA	James	20	NA	NA	Audrey
6	3	1	Dudley	21	8	7	James
7	4	5	Harry	22	8	7	Albus
8	10	9	Ginny	23	8	7	Lily
9	NA	NA	Arthur	24	17	11	Rose
10	NA	NA	Molly	25	17	11	Hugo
11	10	9	Ron	26	18	16	Victoire
12	10	9	Fred	27	18	16	Dominique
13	10	9	George	28	18	16	Louis
14	10	9	Percy	29	20	14	Molly
15	10	9	Charlie	30	20	14	Lucy

ID is a person identifier; maID is mother ID; paID is father ID. NA indicates missing information. See Rowling et al. (2017) to conjure the full source information or Rowling (1997) for a gentle initiation

### Finding Independent Extended Families

When given a dataset like that shown in Table 1, one of the first questions that arises is “Are all these people related in some way or are there separable smaller families that are easier to analyze?”. This question is particularly thorny when dealing with the more typical case of large pedigree registries containing thousands, if not millions, of people. Tracing all the relationships between 30 people is still quite tractable by hand, even if somewhat tedious. Tracing all the relationships between thousands or millions of people is not tractable, even with computers, unless appropriate algorithms are available. When given information like that in Table 1, the first problem is to find independent genetic lines. For the purposes of the present work, we call these independent genetic lines extended families. Although every individual must be related to *some* other individual in the same extended family, that individual is likely not related to *all* individuals in the same extended family. For example, parents are usually not closely related to each other; nonetheless unrelated parents are included in the same extended family.

**Fig. 2:**
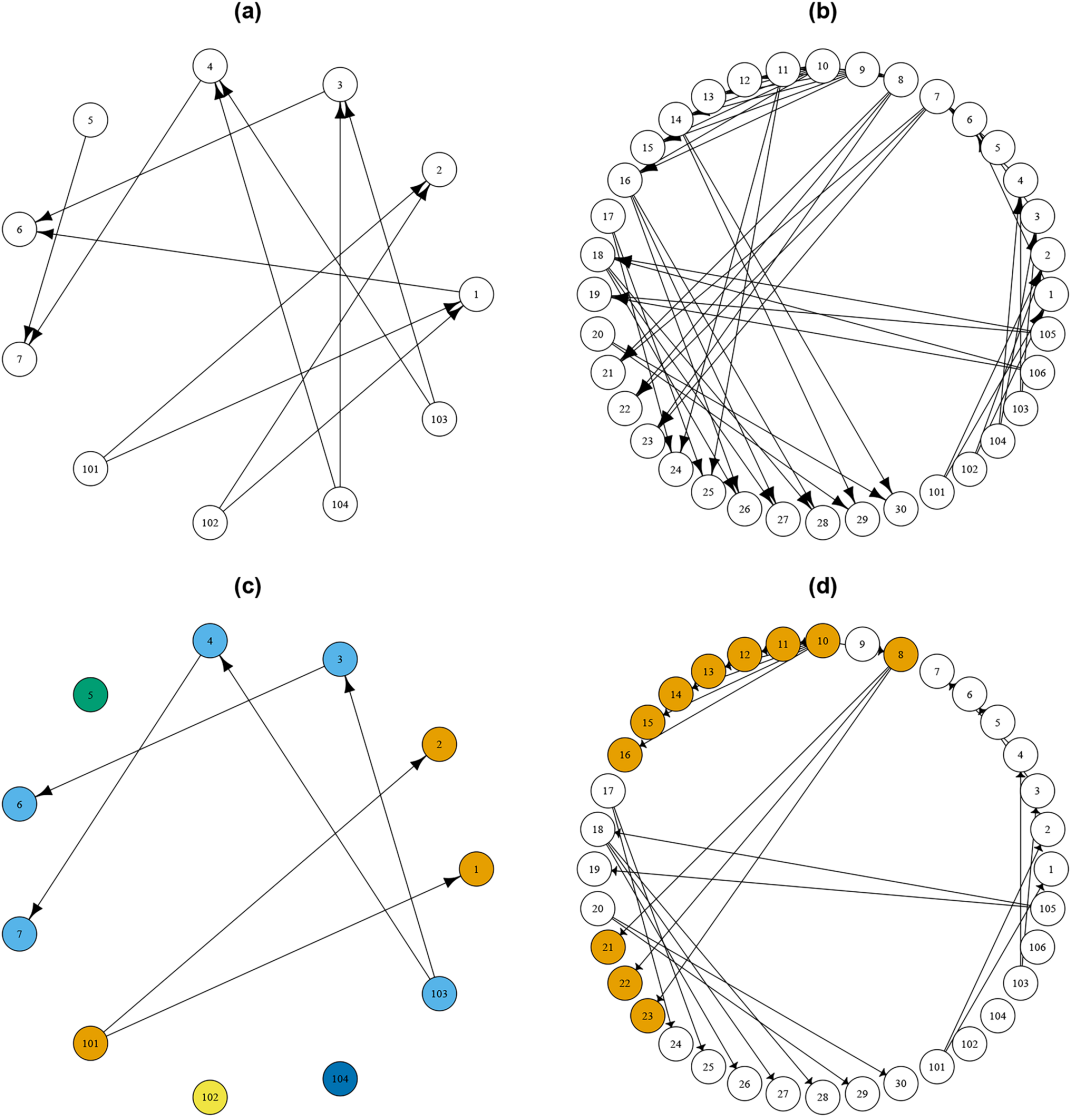
Small example pedigree diagrams as mathematical graphs. Each circular node is a person with node label matching Table 1. In panels (**a**) and (**b**), edges traverse from each parent to their children. In panels (**c**) and (**d**), edges traverse from each mother to each child. **a** A 7-person extended family pedigree with additional nodes for parents that are not fully known. **b** A 30-person extended family pedigree that extends panel (**a**) with an additional generation. **c** A 7-person maternal line pedigree with additional nodes for parents that are not fully known. Nodes of the same color are from the same maternal line. IDs 103, 3, 4, 6, and 7 form one maternal line. ID 5 is its own maternal line. **d** A 30-person maternal line pedigree that extends panel (**c**) with an additional generation. The colored nodes are from the largest maternal line. White nodes are from various maternal lines

The key preliminary step in finding all possible connections between people in a large pedigree is first to turn the pedigree into a mathematical graph. In this graph representation of a pedigree, each person is a node in the graph and parentage produces an edge connecting two nodes. In terms of Table 1, Harry, Lily, and James are each nodes on a graph. Furthermore, because Lily is Harry’s mother, there is an edge connecting the Lily node and the Harry node. So, Lily and James are both connected to Harry; however, Lily and James are not directly connected to each other. Rather, their connection is indirect and is traced through their mutual connection to Harry. Figure 2 shows the graph representation of both the 7-person pedigree and the 30-person pedigree.

To find the extended families in a graph, we must search the connections in the graph. Suppose we start from node 1 in Fig. 2 (a), corresponding to Vernon in Table 1. ID 1 is directly connected to IDs 101, 102, and 6 (Dudley). These are first degree connections to ID 1 (Vernon). We then examine all the direct connections of IDs 101, 102, and 6 (Dudley). These are second degree connections to ID 1 (Vernon). ID 101 and 102 have only one other connection: ID 2 (Marjorie). ID 6 (Dudley) has only one other connection: ID 3 (Petunia). So, the second degree connections are IDs 2 and 3 (Marjorie and Petunia). Now we find the direct connections of IDs 2 and 3 (Marjorie and Petunia). ID 2 (Marjorie) has no new connections, whereas ID 3 (Petunia) is connected to IDs 103 and 104. Thus, the third degree connections are IDs 103 and 104. The only new connection from IDs 103 and 104 is ID 4 (Lily): the fourth degree connection. At this point, the connections become quite sparse: ID 4 (Lily) connects to ID 7 (Harry), and finally ID 7 (Harry) connects to ID 5 (James). From ID 5 (James) there are no new connections, so our search terminates.

We can then collect all the nodes that we visited in this search: IDs 1, 101, 102, 6, 2, 3, 103, 104, 4, 7, and 5. All of these nodes are in the same extended family: there is some degree of relatedness between all of these individuals. Because we visited all the nodes in the graph, we conclude that everyone is in the same extended family. If the search that initiated from ID 1 did not encompass all the IDs in the pedigree database, we would initiate a new search at the first ID that was not already visited. The process then continues until all IDs are partitioned into extended families.

The search proceeded by broadly mapping all possible connections of a given degree before moving to a deeper degree of removal from the ID that initiated the search. We initiated the search at ID 1. So, ID 1 was a zeroth degree connection to itself. From there, the first degree connections were IDs 101, 102, and 6. Then the second degree connections were IDs 2 and 3, which led to the third degree connections for IDs 103 and 104. Finally, fourth, fifth, and sixths degree connections were for IDs 4, 7, and 5, respectively. This search procedure is an example of breadth-first search. By contrast, depth-first search initially traces progressively deeper degrees of removal for single connections before broadening to new connections at the same degree of removal. For instance, a depth-first search would trace from ID 1 to ID 6 to ID 3 to ID 103 to ID 4 to ID 7 and finally to ID 5 before tracing from ID 1 to ID 101. Whether by breadth-first or depth-first search, such an algorithm partitions a set of connected individuals into subsets of individuals with some degree of connection among themselves but no connection across distinct subsets. Such a partitioning is called the weakly connected components of a graph (i.e., Union Find), an algorithm for which was made highly efficient by Galler and Fisher (1964).

### Finding Independent Maternal Lines and Paternal Lines

Finding maternal or paternal lines follows a very similar structure to that of finding extended families. Suppose that by examining Table 1 we want to know if Rose and Ginny follow the same maternal line. Alternatively, suppose we wanted to know if Bill and Rose follow the same paternal line. Even from this very small pedigree, neither answer is particularly clear. The problem becomes exponentially more difficult as the size of the pedigree increases, and the number of people and generations along with it. Fortunately, this problem has a very similar structure to that of finding separate extended families. In essence, we want an extended family exclusively through fathers for the paternal line, and we want an extended family exclusively through mothers for the maternal line.

To find a parental line exclusively along either the mother’s side or the father’s side, we can essentially apply the same algorithm as that used to find separate extended families but to a different initial graph. For the mother’s side, we create a mathematical graph where every person is a node, just as when finding the extended families; however, instead of adding an edge for all parentage, we only add an edge between mothers and their children. So, Harry and Lily have an edge connecting them, but Harry and James do not have an edge connecting them. Now, Lily and James have no connection because their indirect connection through Harry no longer exists in the maternal line graph. Applying weakly connected components to this maternal line graph yields all the separable maternal lines in a pedigree.

The process of finding paternal lines is similar to that for maternal lines. Every person is a node in the graph. Edges are added between every father and child. Applying weakly connected components to this graph yields the separable paternal lines in a pedigree.

Panels (c) and (d) of Fig. 2 show the graph structure for mother-child relationships. As shown in Table 1, ID 101 is the mother of IDs 1 and 2 (Vernon and Marjorie, respectively). Thus, Fig. 2(c) has lines drawn from ID 101 to IDs 1 and 2. The same pattern extends to all the mother-child relationships. Just as weakly connected components applied to panels (a) and (b) for parent-child relationships, the same search algorithms applies to mother-child relationships but with a new meaning attached to the resulting subsets of IDs with any remote connection. When mother-child relationships define the graph, and weakly connected components is applied to that graph of mother-child connections, it finds extended families that are purely defined through maternal relatedness rather than broader parental relatedness. In common parlance, these maternally-defined extended families are maternal lines. Notice that the graph structure for maternal lines is quite different from that of extended families. In particular, the genetic lines show that everyone in this example is connected to everyone else: there is only one, large extended family. By contrast, the maternal lines show there are five separate clusters of nodes with connections in panel (c) of Fig. 2. IDs 101, 1, and 2 form one maternal line. Similarly, IDs 101, 103, 3, 4, 6, and 7 form a separate maternal line with no connection to any other IDs. Some maternal lines are singletons, forming maternal lines with only one individual (IDs 5, 102, and 104). When expanding to the 30-person pedigree, one can see eight maternal lines in panel (d) of Fig. 2.

Just as weakly connected components can find genetic lines using a parent-child graph, and maternal lines using a mother-child graph, paternal lines result from analysis of the father-child graph. In cultures that assign surnames based on the father’s surname (e.g., much of western Europe for the last few centuries), paternal lines are sets of people with the same last name before any subsequent name changes (e.g., due to marriage). Table 2 shows the extended family, maternal, and paternal lines for this small example. In general, once a kind of relationship between individuals is defined as a connection on a graph, the weakly connected components search algorithm can find remote connections among individuals along that kind of connection and partition the individuals into subsets of people sharing those connections. Quantifying the degree of relationship between all individuals is another matter altogether.

**Table 2:** Extended family, maternal line ID, paternal line ID, and generation number for small pedigree dataset

ID	Name	Fam	Mat	Pat	Gen	ID	Name	Fam	Mat	Pat	Gen
1	Vernon	1	1	1	1	16	Bill	1	4	4	2
2	Marjorie	1	1	1	1	17	Hermione	1	6	6	2
3	Petunia	1	2	2	1	18	Fleur	1	7	7	2
4	Lily	1	2	2	1	19	Gabrielle	1	7	7	2
5	James	1	3	3	1	20	Audrey	1	8	8	2
6	Dudley	1	2	1	2	21	James	1	4	3	3
7	Harry	1	2	3	2	22	Albus	1	4	3	3
8	Ginny	1	4	4	2	23	Lily	1	4	3	3
9	Arthur	1	5	4	1	24	Rose	1	6	4	3
10	Molly	1	4	5	1	25	Hugo	1	6	4	3
11	Ron	1	4	4	2	26	Victoire	1	7	4	3
12	Fred	1	4	4	2	27	Dominique	1	7	4	3
13	George	1	4	4	2	28	Louis	1	7	4	3
14	Percy	1	4	4	2	29	Molly	1	8	4	3
15	Charlie	1	4	4	2	30	Lucy	1	8	4	3

ID is a person identifier; fam is extended family ID; mat is maternal line ID; pat is paternal line ID; gen is the generation number with 0 as the first generation. See Rowling (1997) and Rowling et al. (2017) for further illuminating details

### Determining Extended Family Relatedness Coefficients

The previous sections illustrated ways to detect that *some* relationship exists between every pair of people in an extended family, or that a pair of people share the same maternal or paternal line of relatives. However, it is critical for biometric analysis of large pedigrees that we can *quantify* the expected genetic relationship between each pair of people in a pedigree data set. Similarly, although some relationships might exist between a pair of people, it does not necessarily imply that the pair is genetically related. For instance, we know from Table 2 that Dominique and George are in the same extended family, but we want to know if Dominique and George are genetically related at all, and if so, by how much. The previous steps help with the task of quantifying the relationship between each pair of people by reducing the size of the problem. By first finding the independent extended families that have any mutual relationships, we know that we can quantify the amount of genetic relationship separately for each extended family. This approach loses no information because, by definition, an extended family is the set of all people with some relationship to one another. Therefore, there are necessarily no relationships between members of distinct extended families.

The way to quantify the amount of genetic relatedness between (perhaps distantly) related individuals has in principle been known for more than a century (Wright 1922). The original path tracing rules state that (1) the coefficient of relatedness between a mother and child is.5; (2) the coefficient of relatedness between a father and child is.5; (3) to obtain the coefficient of relatedness between any other pair of people, find the shortest paths between the pair and multiply all the coefficients along this path, but sum together paths of the same length; and (4) the shortest paths are found by tracing backwards from one person to the first common ancestor, then tracing forwards to the other person.

Before tackling the more complicated case of Dominique and George, consider the simpler case in Fig. 1 (a) of Harry and Dudley. We start with Harry, and are seeking some common ancestor between Harry and Dudley. Harry has two parents, but James has no ancestors and no direct connection to Dudley so that path terminates. Harry’s other parent, Lily, does have parental information, and these parents are also the parents of Petunia, who is Dudley’s mother. So, there are two shortest paths from Harry to Dudley. One path traces from Harry to Lily to Lily’s father, then down to Petunia and finally to Dudley. Recalling that each connection multiplies the coefficient of relatedness by.5, this path has .5*.5*.5*.5 =.0625. The other path traces from Harry to Lily to Lily’s mother, then down to Petunia and finally down to Dudley yielding .5*.5*.5*.5=.0625. Summing these paths gives 0.125 for the coefficient of relatedness between Harry and Dudley. This coefficient matches our intuitive understanding of relatedness from the knowledge that Harry and Dudley are cousins: their mothers are sisters.

In principle, the same manual path tracing applies to all relationships; however, manual application of path tracing rules quickly becomes complicated. Consider again the relationship between Dominique and George. We can trace Dominique back to George’s brother Bill, and then back to the nearest common ancestors of both George and Dominique, namely Arthur and Molly. So, the shortest paths from Dominique to George travel from Dominique to Bill to Arthur/Molly and then to George, leading to paths of .5*.5*.5=.125. Thus, Dominique and George are have a coefficient of relatedness of .25. Just as with the simpler case of Harry and Dudley, the relatedness of Dominique and George matches our understanding of relatedness from the knowledge that Dominique and George are uncle and niece: George is Dominique’s father’s brother.

An interesting question arises with the case of George and Dominique because – in addition to George having several siblings – George has a twin. How can an algorithm such as that proposed here handle twins? In general, dizygotic (fraternal) twins need no special handling, whereas monozygotic (identical) twins require some adjustments. For the purposes of genetic path tracing, dizygotic twins are just full siblings. By contrast, monozygotic twins are genetically identical, and thus can be handled by keeping only one member of the monozygotic twin pair in the database, and subsequently duplicating all of one twin’s relations to the other twin. Essentially, monozygotic twins are treated as genetically the same person for the purpose of path tracing relatedness patterns.

A final unique situation to consider is adoption. Adoption is irrelevant to the inheritance patterns studied here, which are exclusively genetic. However, special care is needed when interpreting the mother IDs and father IDs that these refer to *biological* mothers and fathers rather than people with primary caregiving roles. Provided that the mother and father IDs refer to biological parentage, no special handling is needed for adoption designs so that appropriate extended families or genetic coefficients can be constructed. There may of course be interest in adoptive parents for the novel modeling possibilities these situations present.

Although any specific relationship coefficient is relatively straightforward to compute, the complication of these computations increases with larger pedigrees. Even the relatively small and simple pedigrees of Fig. 1 would be quite cumbersome for anyone to completely trace by hand. For large-scale pedigree analysis to be practical, an algorithmic approach to these path tracing rules is necessary. Moreover, the path tracing algorithm would ideally compute all relatedness coefficients for a large family simultaneously rather than one at a time, storing them in a two-dimensional array that shows how each person is additively related to every other person. We call such a matrix the additive genetic relatedness matrix, but in animal breeding they refer to it as the numerator relationship matrix (e.g., Emik and Terrill, 1949).

### Determining Relationship Degree and Generation Number

A final critical task for analysis of extended pedigrees is determining the degree of relationship between pairs of individuals, and likewise being able to arrange a pedigree algorithmically into appropriate generations. The degree of relationship between individuals is closely related to the number of paths one must trace to traverse from one person in a pedigree diagram to the other. Relationship degree is a particularly important metric for large, complex pedigrees where less typical relationships exist that make the relatedness coefficient less intuitive (e.g., twins, half-siblings, quarter-cousins, the offspring of a pair of 4th cousins, etc.). Similarly, the generation number allows for intuitive and visual representations of relationships without needing to rely on further information like birth year. The generation number aligns descendants according to the number of unidirectional steps from a target relative: individuals who are the same number of single-direction paths removed from a target relative belong to the same generation. The generation number is especially useful for distinguishing between different kinds of relationships that have the same coefficient of relatedness. For example, parent-child relationships as well as full sibling relationships both have relatedness coefficients of .5, but parent-child relationships are between members of different generations whereas full sibling relationships are between members of the same generation. The same situation occurs for aunt-niece pairs and half-sibling pairs at a .25 relatedness coefficient.

Determining relationship degree and generation number is quite trivial for small pedigrees when presented in standard pedigree diagrams like that of Fig. 1, yet the same task becomes nearly impossible when one must rely solely on the kind of tabular information found in typical pedigree data like that of Table 1. It is not at all clear from Table 1 if James (ID 21) and Percy (ID 14) belong to the same or different generations, or what the length of the shortest path is between these two. From Fig. 1 and Table 2, we can see that James (ID 21) is listed in the fourth generation, whereas Percy (ID 14) appears in the third generation. Likewise from Fig. 1 we can see that James (ID 21) and Percy (ID 14) are second-degree relatives.

For large pedigrees, graphical and visual inspection is not a scalable solution. A mechanism for computing relationship degree and generation number is needed to automate these tasks. In the following sections, we provide computational algorithms that efficiently solve all of the critical pedigree analysis problems thus far discussed.

## Large Pedigree Segmentation into Genetic, Matrilineal, and Patrilineal Lines

When attempting to segment large population databases into a set of smaller extended pedigrees, visual inspection and simple, manual search procedures are insufficient protocols. Fortunately, algorithms from network theory and computer science can solve precisely this problem when the pedigree is constructed appropriately. Once the pedigree is represented as a mathematical graph, we can apply computationally efficient algorithms to search for connections on this graph. In particular, the sets of people that have *any* connection to each other no matter how remote are given by the weakly connected components algorithm (i.e., Union Find) made highly efficient by Galler and Fisher (1964). In essence, this algorithm traces along the graph using depth-first or breadth-first search to partition the sets of nodes into mutually exclusive groups that have at least one (possibly quite remote) connection within the group and no possible connections between the groups. Thus, each group is one extended family. *Mirabile dictu*, the same algorithm solves the related problems of finding maternal lines and paternal lines; only the original mathematical graph that defines connected units must be altered.

The BGmisc package (Garrison et al. 2024) contains the ped2fam(), ped2maternal(), and ped2paternal() functions which add ID variables to a pedigree dataset for the extended family, maternal line, and paternal line, respectively. These functions create graphs based on the input pedigree data file containing person ID, mother ID, and father ID. The graph is then used to generate weakly connected components for the pedigree and append the appropriate ID variable to the original pedigree data file.

Critically, finding weakly-connected components is highly efficient and can be applied to very large datasets. In our experience, when applying weakly connected components to a large dataset with over 700,000 people, the algorithm finished in about two seconds on an ordinary computer (e.g., 16 gigabytes [GB] of random access memory [RAM], 64-bit operating system, 1.80 gigahertz processor, 512 GB solid state drive).

The benefit of first applying weakly connected components to a large pedigree file is that subsequently, we can apply more computationally demanding tasks to the separate extended families. The most computationally demanding of these is finding the average genetic relatedness of each pair of people in a large pedigree database. The BGmisc package (Garrison et al. 2024) implements a fast and efficient algorithm for computing this additive genetic relatedness matrix in the ped2add() function and alternative relatedness patterns in the ped2comp() function, which in turn can be used with the ggpedigree() function in its sister package (ggpedigree; Garrison, 2025; 2026) for plotting family trees.

Broadly, our algorithm for the additive genetic relatedness matrix initially creates an adjacency matrix, notated as $\boldsymbol{A}$, containing first-degree relationships by mapping parents to children. The algorithm then uses this matrix of direct genetic effects to create higher-degree genetic effects: $\boldsymbol{A}^2$ for second-degree effects, $\boldsymbol{A}^3$ for third-degree genetic effects, and so on. The matrix of total genetic effects across all degrees is the sum of zero-degree effects through the highest degree present in the data structure. The total genetic effects matrix is notated as $\boldsymbol{I} + \boldsymbol{A} + \boldsymbol{A}^2 + \ldots = (\boldsymbol{I} - \boldsymbol{A})^{-1}$. This total genetic effects matrix follows the classic path tracing rules of McArdle and McDonald (1984). Finally, this total genetic effects matrix is multiplicatively combined with a self-relatedness matrix $\boldsymbol{S}$ to yield the additive genetic relatedness matrix $\boldsymbol{R}$. Appendix A provides technical details on the mathematical construction of these matrices, whereas Appendix B provides related computational details of its fast, efficient implementation.

The main benefits of the current algorithms over those commonly found in animal breeding (e.g., Quaas, 1976; ter Heijden et al. 1977; Taylor and Tomaszewski, 1989; Nilforooshan et al. 2021; Nilforooshan, 2024) are (1) the novelty of the segmenting algorithm, (2) the implementation of all the algorithms in an open source R package BGmisc, (3) their use of sparse matrices, and consequently (4) their ability to speedily handle extremely large and sparse pedigrees. Although a full performance comparison is beyond the scope of the present work, preliminary testing showed that the BGmisc implementation of the additive relatedness matrix was approximately 66% faster than the fastest method evaluated by Nilforooshan et al. (2021) on their 20,000-person extended family (8.9 seconds versus 14.8 seconds).

Recently, Pedersen et al. (2025) published a related set of algorithms for computing relationship degree using geodesic graph distance (i.e., shortest path between relatives). If $d_{ij}$ is the degree of relatedness between person *i* and person *j*, then $2^{-d_{ij}}$ is the kinship-by-path estimator of the additive genetic relatedness between person *i* and person *j*. As is well-known (Wright 1922; Emik and Terrill 1949; Quaas 1976; ter Heijden et al. 1977; Taylor and Tomaszewski 1989), such a method fails to account individuals with partially known parentage or individuals whose parents are (perhaps distant) genetic relatives. These shortcomings make kinship-by-path estimates useful approximations of additive genetic relationship coefficients, but less robust to the complexities of large, population databases than the algorithms we propose.

## Illustrative Example

**Fig. 3:**
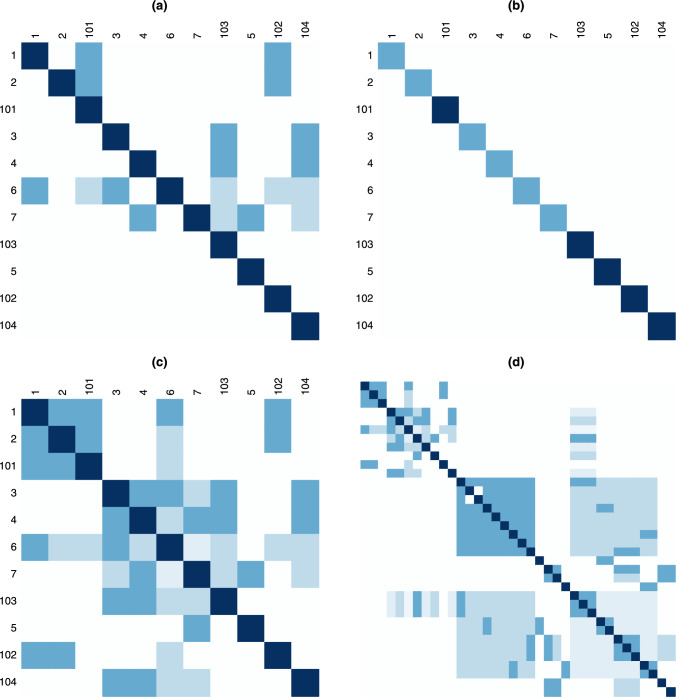
Small example pedigree relatedness matrices. Each row and column is a person. Shading indicates the amount of relatedness with darker shades representing higher relatedness and white representing zero relatedness. Panel (**a**). Panel (**b**). Panel (**c**) has the relatedness matrix corresponding to Fig. 1 (**a**) with IDs matching Table 1. Panel (**d**) has the relatedness matrix corresponding to Fig. 1 (**b**) with the upper left diagonal block containing everyone from panel (**a**) in the same order

To illustrate the capabilities of the tools for path tracing on large pedigrees and extended families, we show the algorithmically computed relatedness matrix for the small example family in Fig. 3 (c), along with its more extended counterpart in Fig. 3 (d). Panels (a) and (b) of this figure show preliminary computational steps to the full relatedness matrix. All panels of Fig. 3 use color to show the amount of relatedness with the darkest shade on the diagonal anchored at unity and white indicating zero. Figure 3 (a) shows the total genetic effects matrix for the small pedigree: $\left( \boldsymbol{I} - \boldsymbol{A} \right) ^{-1}$. Because this small pedigree only contains up to second-degree relatives, in this case $\left( \boldsymbol{I} - \boldsymbol{A} \right) ^{-1} = \boldsymbol{I} + \boldsymbol{A} + \boldsymbol{A}^2$ with all higher powers of $\boldsymbol{A}$ being zero matrices. Moreover, each degree of relation can be seen by the shading of the entries in Fig. 3 (a). The zeroth-degree relatives corresponding to $\boldsymbol{I}$ are the darkest blue diagonals of this figure; the first-degree relatives corresponding to $\boldsymbol{A}$ are the medium blue entries in this figure; and the second-degree relatives corresponding to $\boldsymbol{A}^2$ are the lightest blue entries in this figure. Each part of the total genetic effects matrix can thus be seen as a separate shading in this figure. Figure 3 (b) shows the scaling matrix $\boldsymbol{S}$ which has 1 for each person with no parental information, and 0.5 for each person with complete parental information. Panels (a) and (b) are multiplied according to Equation 3 to create the total additive genetic relatedness matrix which is shown in Fig. 3 (c). A worked example that shows the various steps of matrix multiplication is available at https://cran.r-project.org/web/packages/BGmisc/vignettes/v0_network.html.

Figure 3 (c) shows the full relatedness matrix of the pedigree in Fig. 1 (a). In animal behavior, this matrix is called the numerator relationship matrix (Quaas 1976). Figure 3 (c) groups into clear clusters of people with large amounts of relatedness within a cluster, but somewhat less between clusters. The first cluster is for Vernon, Vernon’s sister Marjorie, and their mother. The second cluster is for Petunia, her sister Lily, and both of their children, Dudley and Harry. The relatedness matrix is arranged into clusters by maternal lines. Comparison to Fig. 2 (c) makes these clusters more evident by looking for groups of nodes with any connection to one another. Fig. 1 (a) similarly shows clustering by maternal lines.

Figure 3 (d) shows the full relatedness matrix of the pedigree in Fig. 1 (b). The first cluster in the upper left region of the relatedness matrix is identical to the entirety of Fig. 3 (c). The second cluster contains Arthur and Molly along with their children. Finally, Arthur and Molly’s grandchildren primarily appear in the lower right block.

A feature of many empirical extended family relatedness matrices is their sparsity. Even though – by definition – every person in an extended family has at least some genetic link to at least one other person in the extended family, there often appear to be numerous pairs of people with zero genetic relatedness. A common way for this lack of genetic relationship to occur is via shared parentage. Typically, parents have no genetic link to one another, but are both linked to their mutual children. For example, IDs 3 and 1 (Petunia and Vernon) are not genetically related to each other, but are in the same extended family because both are genetically related to ID 6 (Dudley). A similar situation occurs with relatives “by marriage”^1^. IDs 1, 2, 101, and 102 (Vernon, Marjorie, and their parents) are a highly genetically interconnected, but they are not genetically connected to ID 5 (James). They are in the same extended family strictly because of relations “by marriage” through ID 3 (Petunia).

Another feature of many empirical extended family relatedness matrices is their broadly blockwise structure. These blocks are most frequently created by the smaller nuclear families that compose the extended family. The children of Arthur and Molly in the second block of Fig. 3 (d) are a clear example of a nuclear family block.

By defining other kinds of relatedness patterns, it becomes possible to represent these more nuanced features of extended families. In particular, full siblings create an important set of relationships within extended families. The BGmisc package provides a ped2cn() function that creates relatedness matrices for “common nuclear” environments that are defined by the distinct sets of full siblings. This full sibling pattern of relatedness complements the additive genetic relatedness found by path tracing rules and the maternal and paternal lines previously discussed. Although the primary aim of the present work is not to discuss modeling of extended families, the novel kinds of relationships extended families present afford numerous opportunities for developing and testing new mechanisms that explain the similarities and differences between people in large population databases.

## Discussion

Although large population databases are not uncommon, the complex pedigrees contained within these databases are far from fully utilized. The complexities of these pedigrees are a double-edged sword: they present exciting opportunities for new intergenerational and biometric research questions and designs while simultaneously stifling the answers to these questions by their very complexity. We have outlined two classes of challenges that large population databases present, and provided conceptual solutions to them. Furthermore, we have implemented these solutions in open-source software in the BGmisc package within the R programming language.

The two classes of challenges were (1) partitioning large population databases into independent segments that can be treated independently, and then (2) quantifying relationships within each of those independent segments. The partitioning problem was solved by first representing the population database as a mathematical graph and then finding the weakly connected components (i.e., Union Find) on that graph. The quantification problem was solved by reliance on Wright’s (1922) and McArdle’s and McDonald’s (1984) linear algebra for path tracing rules initially devised for pedigrees and subsequently generalized for structural equation models. The linear algebra for these path tracing rules is sufficiently general to automatically handle an immense variety of extremely large extended family structures, and even account for genetic bottleneck effects from small founder populations.

Once solutions for these general problems are available, a set of closely related problems are readily solved. The problem of segmenting into other kinds of family lines like maternal and paternal lines follows the exact same structure as that of finding independent genetic lines (i.e., extended families). Similarly, the problem of finding the degree of relatedness is closely related to the linear algebra for the path tracing rules. Moreover, determining the hierarchical organization of an extended family into generations is closely akin to that of organizing a structural equation modeling diagram via the RAMPath algorithm (McArdle and Boker 1990; Boker et al. 2002).

Critically, all of the solutions presented in this work rely exclusively on a supremely limited amount of information: child ID, mother ID, and father ID. Using only that minimal information, a population database can be segmented into independent extended families with separate maternal and paternal lines, organized into generations, given degrees of relationship between all pairs of individuals, and quantified by additive genetic similarity. Extremely limited information is required for these critical tasks. Furthermore, the same graph-theoretic segmentation algorithms and linear algebra path tracing rules can be applied to any set of connections or kinds of relatedness in a pedigree. The generality of these techniques afford manifold possibilities for researchers to explore new avenues of scientific discovery on large population databases.

In spite of these solutions, there remain important limitations and further challenges with working with large population databases. Data cleaning is paramount among these remaining challenges. Because each of these algorithms intensely relies on the correspondence between the child, mother, and father ID variables, they are sensitive to misspecifications and missing data on these variables. Incorrectly entering an ID variable or not having information on a parent ID variable dramatically reduces the utility of the algorithms presented in this work. Beyond mistakes in data entry, sometimes the nominal parents of an individual are not the biological parents of that individual. Mistaken paternity may occur at a base rate as high as 1%−5% (Guerrini et al. 2022), with the rate of mistaken maternity being nearly but not exactly zero. Because of its prevalence, mistaken paternity casts doubt on the utility of genetic effects along paternal lines that persist over numerous generations. Importantly, however, the social effects of nominal paternity (e.g., status carried by last name, inherited wealth, etc.) apply regardless of genetic paternity. Further effort is needed to produce validation metrics for both population databases and the output of the present algorithms to assure researchers that the data and the constructed graphs are not corrupted by the pernicious influences of these errors.

Beyond the complications of data integrity, there are also obstacles with regard to data size. It seems likely that many large population databases will be effectively composed of a single, giant family. To some extent, this feature may be a property of human familial relationships the same way that these “small world” (Travers and Milgram 1969) networks occur across a wide variety of settings (see Barabási, 2014, for a popular review). Such a network structure wherein almost everyone has at least some connection to someone else greatly limits the usefulness of a segmenting algorithm for finding independent extended families. Moreover, extremely large extended families become computationally intractable for a host of research-related tasks ranging from storing the data, to obtaining basic summary statistics and plots, to sophisticated modeling. However, genetically-informed patterns of relatedness other than standard, additive relatedness remain useful even for databases dominated by a small number of large, extended families. The structure of maternal lines, paternal lines, and clusters of full siblings prohibit the kind of sprawling networks that often occur in extended families. Moreover, not all pedigree databases are based on freely breeding, complete populations like national registries. The animal behavior literature frequently analyzes populations subjected to planned breeding programs (van der Werf 2009). Furthermore, pedigree databases occur across a wide array of purposefully incomplete, random probability sampling designs. For example, the United States National Longitudinal Survey of Youth is a national household probability sample with tens of thousands of individuals, and has a mean extended family size of about eight people (median 3 people; Rodgers et al. 2016). Parsing a large database into extended families is much more useful in these sampled designs.

Future work must develop a suitable suite of analysis methods that can tackle the complexities of population databases and extended families. Ready analysis methods for these extremely large and complex data are clearly needed. Standard approaches using conventional structural equation modeling techniques are not suitable for the large-dimensional data encountered in this setting. The standard structural equation model for a single outcome variable on twin data has one variable for each twin, thus creating a *2 × 2* expected covariance matrix with 3 unique elements. Direct application of the same method for a relatively small database of 5,000 people would yield a *5,000 × 5,000* covariance matrix with over 12 million unique elements (12,502,500 elements to be precise). A structural equation model of this size is simply not tenable. However, if the database can be partitioned into thousands of families each of a more modest size – say, 10–100 people per extended family – then a structural equation modeling approach remains entirely feasible in a sufficiently flexible program like OpenMx (Neale et al. 2016).

Methods developed for single, large families might also be plausible alternatives. Genome-wide complex trait analysis (Yang et al. 2011) or a multivariate extension of this (Kirkpatrick et al. 2021) both could address the kind of modeling needs required by large population databases. However, these single-family methods may need multiple family extensions as multilevel models (Pritikin et al. 2017; Hunter 2021; McArdle and Prescott 2005; Shor et al. 2019), and the conceptual difficulties of choosing appropriate hypotheses suitable to these data persist. For example, even the Nuclear Twin and Family Design of Keller et al. (2009) – a design with just four people per extended family – allows considerably more variance components and intricate relations among these components than a classical twin design.

A great benefit of extended pedigree designs is their ability to identify novel variance components. Landmark work by Eaves, Last, Young, and Martin (1978, p. 281–301) showed the numerous unique research questions afforded by extended pedigrees, including new ways to understand environmental effects and gene-by-environment interactions. Looking beyond twin data to data on twins and their parents already opens novel questions for scientific inquiry: for instance, questions related to phenotypic assortative mating versus mate selection on broad social background (Eaves et al. 1989; Heath and Eaves, 1985; see Neale and Cardon, 1992, Ch. 17 for a brief overview focused on model specification). In large population databases a considerable variety of variance components can be estimated (e.g., Lyu et al. 2025). In addition to standard variance components corresponding to additive genetic, dominance, common environmental, and unique environmental variance components – which can all be simultaneously estimated in many extended pedigree designs – Hunter et al. (2021) provided a small sampling of further possibilities. Rater or data collector effects can be estimated to account for known sources of nuisance variance. Geographic region or spatial proximity effects can be estimated to account for variance due to migration and settlement patterns (Tamimy et al. 2021). Neighborhood effects can be estimated to account for broader shared environments beyond the rearing environment. Moreover, each of these novel effects could be moderated by one another or by the standard, biometric variance components. The list of new potential variance components afforded by extended pedigrees is only limited by the ingenuity of the research team and the availability of the data to determine these components. In sum, the solution to large pedigree segmentation and relatedness determination opens new ways to capitalize on these rich data sources for further theory development.

## Data Availability

No datasets were generated or analysed during the current study.

## Appendix A: Computing Extended Family Relatedness Coefficients, Relationship Degree, and Generation Number

The foundational insight that allows computation of relatedness coefficients for extended pedigrees is an old one. Sewall Wright’s original path diagrams (1920), coefficients of relationship (1922), and method of path coefficients (1934) form the foundation for calculating relatedness coefficients on extended pedigrees. Wright’s path tracing rules for extended families slowly left their original context and generalized to path tracing rules between observed variables. Essentially, the path tracing rules for extended families and the path tracing rules for structural equation modeling (SEM) are the very same rules. McArdle and McDonald (1984) put these path tracing rules for structural equation models on a firm mathematical foundation in the reticular action model (RAM) form of a structural equation model^2^.

The RAM form of an SEM is fundamentally a graph-theoretic perspective on SEM. Variables are nodes in the graph; edges are paths between variables. In Wright’s (1920) original formulation of path tracing, nodes were people, or more generally organisms; paths between nodes were lines of inheritance. For pedigree path tracing, we return to this original formulation.

To create a modern version of path tracing rules, we only need a subset of the full set of RAM matrices. The full RAM specification has $\boldsymbol{A}$, $\boldsymbol{S}$, $\boldsymbol{F}$, and $\boldsymbol{M}$ matrices (see e.g., McArdle and McDonald, (1984), for the full specification). For path tracing on pedigrees, we only need $\boldsymbol{A}$ and $\boldsymbol{S}$. The $\boldsymbol{A}$ matrix contains asymmetric, unidirectional influences: here inheritance. The $\boldsymbol{S}$ matrix contains symmetric, bidirectional influences: here a kind of self-relatedness. Combining these two matrices appropriately yields the full set of relatedness coefficients for an extended family. Both the $\boldsymbol{A}$ and $\boldsymbol{S}$ matrices have one row for each person in the extended family and one column for each person in the extended family. The $\boldsymbol{A}$ matrix contains the direct relatedness coefficient from the person in column *j* to the person in row *i*. Stated another way, the $\boldsymbol{A}$ matrix contains an entry of .5 if the person in column *j* is the parent of the person in row *i*, and is zero otherwise. In essence, the $\boldsymbol{A}$ matrix defines parentage. The $\boldsymbol{S}$ matrix contains a kind of self-relatedness. Thus, the $\boldsymbol{S}$ matrix is strictly diagonal with 1 s on the diagonal for all people in the extended family who do not have parents also in the extended family (i.e., no parent information). For all people with both parents in the extended family, the $\boldsymbol{S}$ matrix contains a .5 on the diagonal. For people with one parent in the extended family, the $\boldsymbol{S}$ matrix contains a .75 on the diagonal. In pedigree path tracing, the $\boldsymbol{S}$ matrix is a kind of scaling factor that assures everyone’s final computed self-relatedness is 1, whereas the $\boldsymbol{A}$ matrix represents a kind of graph-theoretic adjacency which defines neighboring nodes in a network.

Once the extended family data is transformed into the RAM $\boldsymbol{A}$ and $\boldsymbol{S}$ matrices, the remaining task is to use the RAM linear algebra to create the total covariance matrix between all people. This total covariance matrix gives the relatedness coefficients for every pair of individuals in the extended family. Computation of the total covariance matrix instantiates the exact path tracing rules as a single linear algebra equation.

Recall that the pedigree path tracing rules state that the relatedness coefficient between two people is found by first tracing backwards from one person to the nearest common ancestor, then turn around, and trace forwards to the other member of the pair. One multiplies each step in this route by the direct relatedness coefficient between people at each step (i.e., by .5). Finally, one sums paths of the same length. These steps are simple to explain, but somewhat misleading. The actual pedigree path tracing rules do not stop at the nearest common ancestor, but rather sum across *all* possible paths between two people. These rules are instantiated as the following linear algebra expression for the total covariance matrix given by (McArdle and McDonald 1984, Eq. 8, p. 235):

1 $$\begin{aligned} \boldsymbol{R}= &  \boldsymbol{I} \boldsymbol{S} \boldsymbol{I}^{\mathsf{T}} + \boldsymbol{I} \boldsymbol{S} \boldsymbol{A}^{\mathsf{T}} + \boldsymbol{I} \boldsymbol{S} (\boldsymbol{A}^2)^{\mathsf{T}} + \boldsymbol{I} \boldsymbol{S} (\boldsymbol{A}^3)^{\mathsf{T}} + \ldots \\ &+   \boldsymbol{A} \boldsymbol{S} \boldsymbol{I}^{\mathsf{T}} + \boldsymbol{A} \boldsymbol{S} \boldsymbol{A}^{\mathsf{T}} + \boldsymbol{A} \boldsymbol{S} (\boldsymbol{A}^2)^{\mathsf{T}} + \boldsymbol{A} \boldsymbol{S} (\boldsymbol{A}^3)^{\mathsf{T}} + \ldots \nonumber \\  &+ \boldsymbol{A}^2 \boldsymbol{S} \boldsymbol{I}^{\mathsf{T}} + \boldsymbol{A}^2 \boldsymbol{S} \boldsymbol{A}^{\mathsf{T}} + \boldsymbol{A}^2 \boldsymbol{S} (\boldsymbol{A}^2)^{\mathsf{T}} + \boldsymbol{A}^2 \boldsymbol{S} (\boldsymbol{A}^3)^{\mathsf{T}} + \ldots \\ & + \boldsymbol{A}^3 \boldsymbol{S} \boldsymbol{I}^{\mathsf{T}}+ \boldsymbol{A}^3 \boldsymbol{S} \boldsymbol{A}^{\mathsf{T}} + \boldsymbol{A}^3 \boldsymbol{S} (\boldsymbol{A}^2)^{\mathsf{T}} + \boldsymbol{A}^3 \boldsymbol{S} (\boldsymbol{A}^3)^{\mathsf{T}} + \ldots \nonumber \\ & +  \ldots \end{aligned}$$

where $\boldsymbol{R}$ is a square, symmetric matrix that gives the relatedness coefficients between every pair of people in the extended family, the superscript on the matrix $\boldsymbol{A}$ indicates repeated matrix multiplication (not element-wise multiplication), $\boldsymbol{I}$ is an appropriately sized identity matrix (1s on the diagonal and zero elsewhere), and the superscript ^T^ indicates the matrix transpose. In terms of path tracing, the matrix transpose means tracing paths backwards. The “...” in Equation 1 means that the same pattern continues infinitely. So, in theory these sums are infinite. In practice, the structure of the $\boldsymbol{A}$ matrix that defines first-degree relatives means that the sums always terminate in a finite number of steps. The sums terminate when there are no higher-degree relatives to be found.

Two equivalent and more compact expressions for Equation 1 follow:

2 $$\begin{aligned} \boldsymbol{R}= & (\boldsymbol{I} + \boldsymbol{A} + \boldsymbol{A}^2 + \boldsymbol{A}^3 + \ldots ) \boldsymbol{S} (\boldsymbol{I} + \boldsymbol{A} + \boldsymbol{A}^2 + \boldsymbol{A}^3 + \ldots )^{\mathsf{T}} \end{aligned}$$

3 $$\begin{aligned} \boldsymbol{R}= & \left( \boldsymbol{I} - \boldsymbol{A} \right) ^{-1} \boldsymbol{S} \left( \left( \boldsymbol{I} - \boldsymbol{A} \right) ^{-1} \right) ^{\mathsf{T}} \end{aligned}$$

The equivalence of Equations 2 and 3 can be verified by showing that

4 $$\begin{aligned} & (\boldsymbol{I} + \boldsymbol{A} + \boldsymbol{A}^2 + \boldsymbol{A}^3 + \ldots ) \left( \left( \boldsymbol{I} - \boldsymbol{A} \right) ^{-1} \right) ^{-1} \nonumber \\ & = (\boldsymbol{I} + \boldsymbol{A} + \boldsymbol{A}^2 + \boldsymbol{A}^3 + \ldots ) \left( \boldsymbol{I} - \boldsymbol{A} \right) \end{aligned}$$

5 $$\begin{aligned} & = \boldsymbol{I} + \boldsymbol{A} + \boldsymbol{A}^2 + \boldsymbol{A}^3 + \ldots - \boldsymbol{A} - \boldsymbol{A}^2 - \boldsymbol{A}^3 - \ldots = \boldsymbol{I} \end{aligned}$$

which implies that $(\boldsymbol{I} + \boldsymbol{A} + \boldsymbol{A}^2 + \boldsymbol{A}^3 + \ldots ) = \left( \boldsymbol{I} - \boldsymbol{A} \right) ^{-1}$. This equality holds whenever $\boldsymbol{A}^p = \boldsymbol{0}$ for some finite matrix power *p*. In general, each matrix power of $\boldsymbol{A}$ represents a degree of relatedness. $\boldsymbol{A}^0 = \boldsymbol{I}$ represents zero-th degree relatedness, or self-relatedness. $\boldsymbol{A}=\boldsymbol{A}^1$ represents first-degree relatedness, or parentage. $\boldsymbol{A}^2$ represents second degree relatedness, or grandparentage, and so on.

Although Equations 1–3 are all equivalent, they facilitate different interpretations. Equation 1 most clearly aligns with the definition of path tracing rules. In Equation 1 there is a sum of several terms. Each one of these terms is the relatedness from one kind of path. The total relatedness between people is the sum over all possible paths. The first term in Equation 1, $\boldsymbol{I} \boldsymbol{S} \boldsymbol{I}^{\mathsf{T}}$, reads as follows^3^: trace backwards zero times from the current person ($\boldsymbol{I}^{\mathsf{T}}$), turn around ($\boldsymbol{S}$), then trace forwards zero times from the current person ($\boldsymbol{I}$). Thus, the first term only concerns self-relatedness. The third term in the second line of Equation 1 reads analogously as follows: trace backwards for two generations ($(\boldsymbol{A}^2)^{\mathsf{T}}$), turn around ($\boldsymbol{S}$), then trace forwards for one generation ($\boldsymbol{A}$). Thus this term represents aunts and uncles being related to their nieces and nephews. Theoretically, this path tracing sums infinitely many possible paths. Practically, this path tracing always terminates in a finite number of paths. The total relatedness between any pair of people is the sum over all such possible paths.

In structural equation modeling, $\boldsymbol{A}$ represents the direct effects, whereas $\left( \boldsymbol{I} - \boldsymbol{A} \right) ^{-1}$ represents the total effects. By analogy in pedigree path tracing, $\boldsymbol{A}$ represents direct genetic effects: inheritance by direct parentage, and $\left( \boldsymbol{I} - \boldsymbol{A} \right) ^{-1}$ represents the total genetic effects from all relatives. The path tracing rules of structural equation models and pedigrees are – in fact – the same rules when the matrices are appropriately defined.

Equations 1–3 have shown how to compute the extended family relatedness coefficients, but computing relationship degree and generation number are closely related problems that are solved by similar methods. As noted previously, the matrix powers of $\boldsymbol{A}$ contain the degrees of all relatives. Hence, the nonzero elements of $\boldsymbol{A}$ contain all the first-degree relatives. The nonzero elements of $\boldsymbol{A}^2$ contain all the second-degree relatives. If there are any nonzero elements of $\boldsymbol{A}^{25}$, these elements are the 25th degree relatives. For any real data set, there always exists some power *p* such that $\boldsymbol{A}^p = \boldsymbol{0}$. If *p* is the largest value such that $\boldsymbol{A}^p \ne \boldsymbol{0}$, then *p* is the maximum number of generations in the extended family.

Classifying people into generations also relies on the matrix powers of $\boldsymbol{A}$. The generation of a person *i* is the largest power *p* such that the *i*th row of $\boldsymbol{A}^p$ has any nonzero elements. In practice, this generation number is easiest to compute iteratively. Start every person in generation 1. Compute $\boldsymbol{A}^2$. Add 1 to every person’s generation that has any nonzero elements in their row of $\boldsymbol{A}^2$. Repeat this process until $\boldsymbol{A}^p$ is all zeros. By this algorithm, people with fewer ancestors are placed in earlier generations; whereas people with more ancestors are placed in later generations. Virtually the same procedure for sorting people into generations was used by the RAMPath diagramming algorithm to hierarchically arrange variables in SEM diagrams (McArdle and Boker 1990; Boker et al. 2002).

In the BGmisc package, the ped2comp and ped2add functions compute the additive genetic relatedness. Similar functions exist within the BGmisc package for other kinds of relatedness coefficients. For example, a full sibling relatedness coefficient would be unity for each pair of individuals that are full siblings, and zero otherwise. Similarly, a mitochondrial relatedness coefficient would be unity for all pairs of people that share mitochondrial DNA, and zero for all pairs that do not. All of these relatedness coefficients can be computed for any pedigree, but become impractical for large pedigrees. Whenever possible, segmenting a pedigree into smaller extended families is recommended. A practical limit for these functions is approximately 700,000 to 800,000 people in a single extended family, which takes several hours and significant computer memory to run (e.g., 32 gigabytes [GB] of random access memory [RAM]). An 800,000 person extended family may contain up to 320 billion relationships (for an empirical use case, see Burt et al. 2025). Many simple tasks become cumbersome for data of this size and complexity.

## Appendix B: Further Computational Details of Relatedness Coefficients

Although not the core contribution of the present work, several common computational best practices (generally see e.g., Press, 2007) are followed in the implementation of the algorithms that are the topic of the present work. Because the computation of the relatedness coefficients is the most time consuming, it merits some further details about its implementation in the BGmisc package. Full details can be see in the source code for the ped2add and ped2comp functions therein.

Ultimately, the goal is to compute $\boldsymbol{R}$ from Equation 3. Despite this equation including the expression $(\boldsymbol{I} - \boldsymbol{A})^{-1}$, this matrix inverse is never computed. Rather, we compute the finite sum $\boldsymbol{I} + \boldsymbol{A} + \boldsymbol{A}^2 + \ldots + \boldsymbol{A}^p$ where *p* is the smallest positive integer such that $\boldsymbol{A}^p$ is a zero matrix. The $\boldsymbol{A}$ matrix has one row and column for each person. Each element of $\boldsymbol{A}$ gives the direct genetic effect of the person in the column of the matrix to the person in the row of the matrix. Each column of $\boldsymbol{A}$ has as many nonzero elements as that person has children. Each row of $\boldsymbol{A}$ has zero, one, or two nonzero elements: one for each known parent of that person. According to this definition of $\boldsymbol{A}$, the matrix is very sparse. Thus, BGmisc uses sparse matrix storage and operations as defined in the Matrix (Bates et al. 2023) package. In general these sparse matrix operators only store and operate on the nonzero elements of a matrix, thus making matrix operations no longer scale with the size of the matrix but rather with its sparsity. In an extremely exaggerated case, a 100-million by 100-million square matrix with a single nonzero element can be used in matrix operations with a similar efficiency to a 1 by 1 matrix.

The matrix sum is accumulated according to the following procedure:

At the end of this loop, T stores the total genetic effects matrix $(\boldsymbol{I} - \boldsymbol{A})^{-1}$. The parameter max can be set to an *a priori* maximum degree of relatedness or it can be inferred when B is a zero matrix. Because the powers of $\boldsymbol{A}$ increase in sparsity, eventually terminating in a zero matrix, the matrix multiplication becomes more efficient and the matrix storage becomes smaller as the iterations of the loop progress. Taking advantage of the sparsity in $\boldsymbol{A}$ makes this algorithm particularly advantageous for extremely large pedigrees.

For computation, we further factor $\boldsymbol{R}$ as

6 $$\begin{aligned} \boldsymbol{R} = \boldsymbol{C} \boldsymbol{C}^{\mathsf{T}} \end{aligned}$$

where $\boldsymbol{C} = (\boldsymbol{I} - \boldsymbol{A})^{-1} \boldsymbol{S}^{\frac{1}{2}}$ and $\boldsymbol{S}^{\frac{1}{2}}$ is computed as a diagonal matrix with the square roots of the diagonals $\boldsymbol{S}$. Multiplying a matrix by its own transpose is highly efficient, especially when using sparse matrix computations. Standard algorithms for this kind of computation take advantage not only of the sparsity of $\boldsymbol{C}$, but also of the known result that $\boldsymbol{R}$ is symmetric and therefore only the lower triangle needs to be computed and stored. Many of the older algorithms for path tracing can be seen as earlier examples of taking advantage of the sparsity and structure that are characteristic of $\boldsymbol{R}$ (e.g., Quaas, 1976; ter Heijden et al. 1977; Taylor and Tomaszewski, 1989).

In addition to computation of $\boldsymbol{R}$, model fitting often makes it advantageous to compute its inverse, $\boldsymbol{R}^{-1}$. By some elementary linear algebra (e.g. Leon, 2006) one can see that

7 $$\begin{aligned} \boldsymbol{R}^{-1} = (\boldsymbol{I} - \boldsymbol{A})^{\mathsf{T}} \boldsymbol{S}^{-1} (\boldsymbol{I} - \boldsymbol{A}) = \boldsymbol{D} \boldsymbol{D}^{\mathsf{T}} \end{aligned}$$

as long as all the inverses exist and the matrices are conformable, where $\boldsymbol{D} = (\boldsymbol{I} - \boldsymbol{A})^{\mathsf{T}} \boldsymbol{S}^{-\frac{1}{2}}$. Furthermore, in the pedigree context $\boldsymbol{S}$ is diagonal so its matrix inverse ($\boldsymbol{S}^{-1}$) is merely the reciprocal of the diagonals of $\boldsymbol{S}$. This is a matrix inverse albeit a simple one that does not use a general matrix inversion computation. All the same advantages to the sparse matrix approach to computing $\boldsymbol{R}$ similarly extend to $\boldsymbol{R}^{-1}$. In fact, it is often easier and more efficient to compute $\boldsymbol{R}^{-1}$ than to compute $\boldsymbol{R}$.

## Footnotes

1 Legal marriage is irrelevant here. The relevant factor is mutually produced offspring (i.e., co-parentage). If “marriage” is co-parentage, then relatives “by marriage” are genetic relatives of a co-parent.
